# What do emergency medicine and burns specialists from resource constrained settings expect from mHealth-based diagnostic support? A qualitative study examining the case of acute burn care

**DOI:** 10.1186/s12911-018-0647-1

**Published:** 2018-08-01

**Authors:** Iona Crumley, Lisa Blom, Lucie Laflamme, Helle Mölsted Alvesson

**Affiliations:** Department of Public Health Sciences, Karolinska Institutet, Tomtebodavägen 18A, SE-171 77 Stockholm, Sweden

**Keywords:** Emergency medicine, Remote consultation, South Africa, Burns, mHealth, Qualitative research

## Abstract

**Background:**

Traumatic injury is a serious global health burden, particularly in low- and middle-income countries where medical care often lacks resources and expertise. In these contexts, diagnostic telemedicine could prove a cost effective tool, yet it remains largely underused here, and knowledge on its potential impact is limited. Particularly scarce is the view of the expert user physicians, and how they themselves relate to this technology.

**Methods:**

This qualitative study investigated tele-experts’ (*n* = 15) views on the potential for image based teleconsultation to be integrated in trauma and emergency care services. A semi-structured interview guide was used to gather data concerning an mHealth app for burns diagnostics in the acute care setting, in the Western Cape, South Africa. Questions examined challenges and opportunities in user acceptance and outcomes, in specific case management and in the wider healthcare system. Resulting data were subject to qualitative content analysis.

**Results:**

Experts perceived remote diagnostic support through mHealth as linking directly to several key ideas in medicine, including barriers to care, medical culture and hierarchy, and medical ethics within a society. Ideas running through the data pertained to the widening and narrowing of inherent gaps in the healthcare system, and the formalisation of processes, practices and relationships, effected by the introduction of an app. Wide consensus was stated on positive outcomes such as increased education opportunities, improved professional relationships and a better ability to advise and diagnose, all further facilitated through greater ease of access. The belief was that these could achieve a narrowing of systemic divides within healthcare, although it was acknowledged that the possibility to induce the opposite effect also arose. Differing opinions were voiced relating to the involvement of allied health professionals and feedback.

**Conclusion:**

Experts see several aspects to an mHealth app for remote diagnostic support which could enhance provision of trauma and emergency care in a resource poor setting, relating to reduced delays, streamlined care and improved outcomes. Attention is also drawn, however, to specifics of the environment which would demand further and careful consideration for success – time pressure, intensity and the wide range of subspecialties to be considered.

**Electronic supplementary material:**

The online version of this article (10.1186/s12911-018-0647-1) contains supplementary material, which is available to authorized users.

## Background

Traumatic injury is responsible for more than five million deaths each year, and accounts for around 11% of Disability-Adjusted Life Years (DALYs) globally [1]. Low- and middle-income countries (LMICs) currently bear an unequal brunt of this burden; estimates suggest that up to 90% of deaths due to injury occurs in LMICs, and cases may continue to rise as socio-economic transitions continue apace [2]. Trauma and emergency medicine in these contexts is subject to many challenges typical of health care systems in LMICs, overburdened, and constrained by limited financial, human and physical resources.

Specific characteristics which complicate trauma and emergency cases can also be exacerbated in such settings, such as the following three which are critical for both research and intervention. *Remote Presentation*; Often the initial presentation of these cases is outside of the emergency department, to poorly resourced or remote facilities, a situation compounded by a lack of pre-hospital emergency services [3], and inadequate road and transportation systems [4]. Where specialists exist they are often clustered in major cities, thus expert knowledge is not widely dispersed. *A Vast Array of Cases;* Emergency Medicine (EM), a well-developed specialty itself, entails interaction with all medical specialties [5], as cases are diverse in cause and consequence. *The Time Critical Factor;* in trauma and emergency medicine, every second is critical, and immediacy of care has repeatedly been shown to be paramount [5]. Increased response time, or hesitance in diagnosis and initiating treatment, may lead to diminished outcomes for the patient.

Telemedicine, ‘the delivery of health care services, where distance is a critical factor, using information and communication technologies...’ [6] has the potential to help address these challenges. Studies from high income countries (HICs) show a positive correlation between use of diagnostic telemedicine and reduced patient time in the emergency room, reduced time to transfer to specialist services, reduced costs incurred by the patient or health service and fewer medical errors [7–9]. Diagnostic tools much relied upon in the emergency environment such as Computed Tomography, Plain Film Radiography and Echocardiography are well suited to electronic transfer for remote assessment [10], and studies into burns show that diagnosis can be effectively made through transmitted images reducing both patient transfers and costs associated with treatment [11–13]. Research suggests that interpersonal connections facilitated by telemedicine can diminish professional isolation [14, 15], improve communication, and build professional relationships. Further, an educational benefit is perceived in the exchange of advice and information between very experienced doctors and more junior colleagues [16–21].

mHealth, a sub-segment of telemedicine involving ‘medical and public health practices supported by mobile devices such as mobile phones…’, has seen substantial growth in recent years [22]. As developments in mHealth further explore the potential of smartphone technology, such devices become more affordable, available and applicable in the resource limited context [13, 23]. With these factors in conjunction, diagnostic mHealth could reap similar benefits in outcomes for patients and physicians in low resource settings, as those previously demonstrated by telemedicine in HICs.

As with the introduction of any intervention or technology into a medical setting however, proving its efficacy is only one part of the challenge for adoption into practice. For mHealth, issues to consider for implementation include telecommunications policy, confidentiality of patient data, and financing [1, 24], as well as complex behavioural barriers regarding the motivation to accept a new way of work [24, 25]. Research abounds into both telemedicine, and more specifically mHealth, both quantitative, and, to a lesser extent, qualitative. However, to the best of our knowledge there is little research which explores diagnostic mHealth for acute care in a resource limited setting [26–28]. A knowledge gap was thus identified relating to diversity of situations where mHealth may be applicable, acceptance and outcomes for users, strategies for successful introduction and implementation and the wider challenges and benefits to patients and the health care system.

Further to this, an additional knowledge gap was identified related to expert users’ perspectives in remote advice technology. Diagnostic telemedicine usually implies a three way interaction involving patient, point-of-care (POC) user physician, and remote tele-expert, of whom the latter appear less represented in existing research. As users, these specialists’ experiences of such systems are key, further, they are a great resource regarding the detailed medical specifics which must be addressed by these technologies, because of their vast knowledge and experience.

The study aim was thus to understand expert users’ perceptions of impacts, challenges and opportunities of an mHealth clinical diagnosis system prior to implementation for acute burns in a resource constrained setting, and the specific implications for diagnostic trauma and emergency telemedicine within this context.

## Methods

As a large middle-income country, South Africa faces many challenges in health care at the present time, and is reported to be suffering a quadruple burden of disease - violence, AIDS/HIV, infectious diseases and non-communicable diseases [29]. While excellent health care exists, the public system, which covers up to 84% of the population, suffers from chronic underfunding and shortages, and often struggles to cope with demand [30]. Access to health care is thus limited in a population of 56 million inhabitants, many subject to high levels of poverty, unemployment and limited education, and where much of the population inhabits rural areas poorly served by existing health care facilities [29].

The burden of traumatic injury in South Africa is extensive, with approximately a third of admissions to the emergency department due to injuries, a figure far higher than in many other countries [29]. Of these cases, a substantial proportion is burn injuries; estimates suggest that 3.2% of South Africa’s population suffer burns annually, with burns injuries the third highest cause of injury fatality in the under 18 s. In adults, assault is responsible for the highest percentage (37%) of injuries, followed by accidents, shack fires, and stoves; in children, burns relate more to accidents in the home [31]. In both age groups, consequences of high levels of poverty such as overcrowded housing, unsafe cooking facilities, fuel and power supply are without doubt exaccerbatory factors. [31]

*‘mHealth for Burn Diagnostics and Care in South Africa’* is one telemedicine project, specifically developed to help improve outcomes for acute burns patients, through its deployment in the trauma and emergency setting [32]. Using smart phone technology, the project involved the development of an app which allows doctors in rural, resource poor areas, or secondary level emergency departments, to send images and textual descriptions of burns cases to an on call tele-expert, who then replies via the app with management and referral advice. The mHealth app had been tested and was about to be implemented in a pilot phase across eight hospitals within the Western Cape Province. By conducting interviews prior to implementation, we hoped to take the opportunity to explore perceptions of a telemedicine system in a hypothetical context, allowing for examination unclouded by the particular issues of a system currently in use.

The data collected for this study originally related to the care of acute burns injury patients, but for this paper, was analysed through the lens of an emergency care perspective. As upmost medical emergencies with specific treatment requirements, burns injuries embody many of the challenges within EM, and thus this data was considered highly suited to such an investigation.

### Data collection

An extensive literature review was undertaken pertaining to research into existing telemedicine projects and pilots, evaluation and implementation, outcomes and user acceptability [12, 33–35], with themes arising from these works organised into broad categories of physician impact, patient impact and health system impact. Inspiration was originally drawn from the Information Ecologies Framework, whose holistic approach describes ‘*a system of people, practices, values, and technologies in a particular local environment’,* all aspects of which should be considered for successful implementation of new technology [36]. The interview guide (Additional file 1) was developed from the matrix presented in this framework, using broad questions to facilitate open discussion and capture the issues most relevant to the experts. The interview began with descriptions of current working situation and practices, then moved to investigate formal and informal experiences with apps, and finally explored expectations of the current mHealth app. Two pilot tests were undertaken to ensure comprehensibility and content validity.

Purposive sampling was used to identify the sample, thus physicians working in the Western Cape Province who were qualified to act as tele-experts according to the criteria of the mHealth project were invited to participate. This sample comprised consultant physicians at all stages of their career, and included both emergency medicine and burns medicine experts, allowing representation of the concerns of both specialties. A ‘next generation’ view was sought by also including registrars completing specialist training to become a consultant in either burns or emergency medicine, currently in year 3 or 4 of their training. One further expert participant was included from a different province and unaffiliated to the project in order to gain an outsider perspective, identified by consensus recommendation of several primary interviewees. Of 18 physicians approached to participate in the study, 15 agreed to do so, and written informed consent from each participant was obtained prior to the interview. Participants’ years of experience ranged from 3 to 35 and a range of professional qualification levels was represented. Interviews took place over a period of approximately 2 months. Further details related to the interviews conducted are listed in Table 1; years of experience and professional level have been removed to protect participants’ identities.

**Table 1:** Participant characteristics

ID	Gender	Specialty	Length of interview (in minutes)	Previous knowledge of the app to be implemented
1	M	Emergency Medicine	44	Yes
2	M	Emergency Medicine	79	Yes
3	F	Emergency Medicine	53	Yes
4	M	Burn Surgery	53	Yes
5	F	Emergency Medicine	50	Yes
6	M	Emergency Medicine	90	Yes
7	M	Emergency Medicine	92	No
8	M	Emergency Medicine	95	No
9	F	Burns Surgery	78	Yes
10	M	Burns Surgery	76	Yes
11	F	Emergency Medicine	76	Yes
12	F	Burns Surgery	80	No
13	M	Burns Surgery	56	No
14	F	Burns Surgery	76	Yes
15	F	Burns Surgery	71	No

All participants were interviewed individually using the same interview guide by a team of two interviewers, a UK masters student with a clinical background, and a Swedish PhD student with a public health background. One researcher had been involved in the early phases of the project with project planning, and data collection and presentation to some of the potential expert users. This researcher was therefore known to some participants, but was not one of the project leaders and was not involved in decisions related to development of the app. The second researcher was previously unknown to all participants and had no involvement to the project prior to the study. It was hoped that this team would allow a balance between an ‘insider’ and an ‘outsider’ perspective, and allow the study to benefit from the positives to be gained from these two positions [37]. Ethical approval for this study was granted by Stellenbosch University Health Research Ethics Committee (N15/01/008).

Interviews, lasting between 44 and 95 min were conducted at a time and place chosen by the participant. Recordings were transcribed verbatim or intelligent verbatim, and verified for content by the interviewers. Subsequent to transcription of the material, a summary of topics of each interview was made. Any interviews containing themes which required further clarification or elaboration were identified, and corresponding participants were requested to take part in a brief member check [38].

Every effort was made to enhance trustworthiness of the data [39]. It was hoped that the team of 2 interviewers with differing professional disciplines, experiences, and knowledge of the project allowed two very different approaches to understanding the data. Researchers took care to invest in a process of reflexivity to try to identify any areas in which their previous experiences would cloud the process and adapt for this. Constant discussion between the interview team and wider research team in developing the guide, coding and analysing the data was conducted. Member checks allowed for clarification and uniformity in process for all participants [38], and observational notes allowed us to compare processes as described and as witnessed, such as regarding referral processes.

### Data analysis

The transcriptions, member-check and observational notes were subjected to a qualitative content analysis, driven by the processes described by Graneheim and Lundman [40]. Each interview was listened to and read extensively to allow immersion in the data. Throughout the data set, meaning units were identified, abbreviated to condensed meaning units and allocated a code. After initial coding of the first interview was completed, this was discussed at length among the two interviewers for consensus and amended accordingly. Coding of the full data set was then completed, with continued input and discussion from the wider research team. Codes were studied and collated in sub-categories, and then wider categories. Following this process of organizing and compartmentalizing the manifest ideas, the sense or meanings behind the categories and their associations was explored. From here, two latent themes interconnecting the categories were elucidated. In addition, the material was explored as related to the two distinct specialist groups questioned, and their views on topics were compared for evidence of consensus or disparity.

## Results

**Fig. 1:**
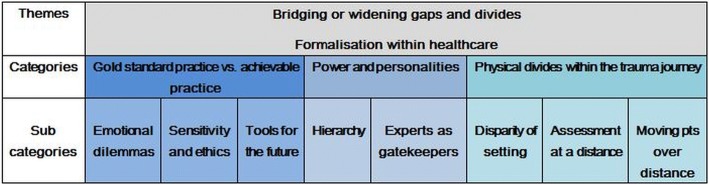
Themes, categories and sub-categories in the content analysis of expert perspectives on the app

In reflecting upon their work, the experts raised the idea of inherent, systemic gaps or divides which hindered their ability, beyond scarce resources, to provide the level of care they idealised. These ‘gaps’ or ‘divides’ were developed as categories in the analysis, and infiltrated many characteristic concerns of trauma and emergency - the immediacy, the inability to refuse a patient, and the emotional and psychological challenges for clinician and patients. The two themes found running through the data were (i) the inherent potential for mHealth to bridge these gaps, or conversely, widen them, and (ii) the formalisation of processes and practices that confer these changes. A summary of the final analysis in table format demonstrating the sub-categories, categories and themes is presented below in Fig. 1, followed by elucidation of the categories identified.

In addition, the different perspectives afforded from the two specialties were investigated, and while there was much coherence in opinion found, the focus of the specialties differed on some key areas.

### Gold standard practice and achievable practice

The first category on “power and personality” included three sub-categories of ‘Emotional dilemmas’, ‘Sensitivity and ethics’ and ‘Tools for the future’, capturing areas in which a discrepancy was highlighted between a ‘gold standard’ practice, and that which was achievable within the realities of the setting (Fig. 1).

#### Emotional dilemmas

In a resource poor setting, and in treating patients with traumatic injuries, there can be a gap between the care health professional's wish to provide for their patient, and what they are able to do in practice, for reasons more diverse than the scarcity of resources. These dilemmas and their emotional and psychological impact came up frequently, both for the experts themselves, and as they envisaged from the perspective of the POC doctors.

*“I think most of these guys, especially at the primary care facilities, urban and non-urban, are really functioning quite a long way beyond where any human being should be functioning to be, sustainably productive” –ID.2*

Pressure to maintain a high turn-over and ‘task fragmentation’ required to address the needs of many patients at once was mentioned as a prominent challenge of the emergency setting. The implications for telemedicine according to the participants were clear; speed, reliability and user friendly design are non-negotiable for success [14, 41]. Such is the relentless intensity of the trauma and emergency field, a system for this arena must be, “*analogous to battlefield apps”.-(ID.2)*, and unreliable service or delayed response could lead to the system being rejected. As one expert described:

*“... in an environment like that, you can’t have long delays, you can’t sort of let the patient lie there while you see if the expert replies. So I think rapid, expert feedback is key, because if people use it once and get back a delayed response, they won’t use it a second time.” –ID.4*

Trauma and emergency can involve treating terrible injuries, whilst dealing with patients in pain, fearful and vulnerable. In addition, the socioeconomic context of some cases can also be emotionally challenging for the medics, where they occur as a consequence of poverty or violence. In the study setting, as described above, domestic abuse and burns resulting from poor housing conditions were commonly reported mechanisms of injury. Speaking of assessing acute burns patients, one expert commented:

*“‘It’s always just that emotional aspect, it’s getting sort of past that, and, sort of the horror of it all, actually having, it’s, I think it’s probably one of the most, em, emotive things that you can see.” –ID.3*

For doctors with limited trauma and emergency experience, often ‘out of their comfort zones’ in terms of subspecialty, the demands of treating these injuries can be overwhelming. In the case of burns, some have only one day’s training before taking up post, often alone.

Some experts expressed a belief that the presence of a senior colleague via an mHealth app, albeit remote, constituted a ‘moral support component’ that could be of comfort to inexperienced doctors. All believed that in taking difficult decisions such as to palliate rather than to resuscitate, expert concurrence was valuable both emotionally as well as medically. Decisive instruction, decision support and trustworthy advice facilitated by interaction with experts was seen as key to limiting inherent pressures within trauma and emergency, whilst not being able to eliminate them.

#### Sensitivity and ethics

All but one expert described how informal ‘telemedicine’ practices were already used extensively, in that images are shared via mobile phone messaging apps in order to consult with colleagues. While confirming the value of image sharing as a concept, some expressed worry around ethical and legal implications in the current system, and saw benefit to a more formalised arrangement. The ability to regulate users for greater security of patient data was one specific advantage listed of such systems, another, the data storage capacity and a legal documentation of having sought help. It was also suggested that using such image records to demonstrate visual improvement to patients, can have a psychological role to play in trauma recovery.

Even in a regulated system however, ethics around photographing injuries, particularly in trauma and emergency where consent may be an issue, are complex. It was suggested that innovations such as apps can exacerbate these issues, making medical practice seem too familiar and removed from the clinical setting.

*“..and so it was easier when you had to dig out your big camera, because it kind of forced you into that cognitive space of being aware that you’re taking pictures of people” –ID.1*

Effective communication with patients and carers is seen as crucial to remedy such problems. One expert suggested ‘pop-up prompts’ within systems, reminding users to explain various stages of the process before use.

#### Tools for the future

Many experts saw education as a vital component of their role, and most viewed telemedicine as an opportunity to enhance their capacity as educators. Teleconsultation’s potential for education is well documented, [21, 42] and has been rated in other studies by POC users as one of the most important aspects to influence their satisfaction in its use [3, 16].

The importance of providing feedback on patient treatment and outcome proved a more divisive issue. Many questioned its necessity to job satisfaction at the expert level, and commented that due to the transient nature of the patient journey through EM, feedback is often inevitably lower priority in this specialty. While some did welcome it, many expressed sentiments such as:

*“for the experts, as heartless as it sounds, no, because I’ve got my own patient load, I’ve got my own patients that I’m dealing with that are complex and that’s enough for me to handle...” –ID.15*

Discrepancies did not lie between specialties, and seemed idiosyncratic or related to personal preference. Other studies have found that expert physicians find patient follow up notifications to be beneficial [25, 43], although these studied experts in HICs providing advice to resource poor settings, potentially acting in a voluntary capacity and through interest or desire for reciprocal learning.

Despite questions raised over the additional workload burden that feedback provision could incur for tele-experts, agreement was complete on its value for POC staff, for their development and encouragement as well as learning opportunities.

Overall, in terms of being able to lift practice from that which was currently achievable to that which was envisaged as gold standard, the potential for telemedicine was believed to relate strongly to educational opportunities, and greater access to support and information, whilst formalising many aspects of technology use already in place through interaction with Whatsapp and other similar services.

### Power and personality

The second category on “power and personality” included two sub-categories both capturing hierarchical dimensions of the health care system (Fig. 1).

#### Hierarchy

Many experts spoke of the hierarchical system that exists within medicine. There was acknowledgment that ingrained systemic fear of seniors could lead to hesitation to seek advice, with subsequent critical delays in patient care, and the effect was imagined to be amplified for nursing and allied health staff. One expert described his first years in medicine:

*“That’s part of medical training, you’re made to feel stupid and there’s always someone smarter than you, so it doesn’t create a culture of openness and asking for help... It was very much the sense I don’t want to disturb anyone, what if my question is stupid, what if they ask me something I don’t know.”- ID.11*

Telemedicine’s possible contributions in spanning these professional divides included its potential anonymity, reducing anxiety in calling, and an allocated expert per shift reducing the sense of ‘bothering someone.’ One expert clarifies:

*“I think it will probably just help in building more positive relationships, because it’s another way of saying we are reachable, you can approach us and I think that helps a lot for doctors who are on the periphery dealing with major problems.”-ID.10*

From the expert level, it was proposed that an app could incorporate an allocated ‘shift system’ – with specific time slots during which an assigned expert was responsible for queries directed to them thought the app. This was viewed as potentially more psychologically manageable than the ‘endless on call’ of an informal system, making experts more amenable to queries, and improving communication as a result.

Both EM and burns experts were split in their opinion on whether nursing staff should also be able to seek advice through such a system. Some thought their inclusion to be crucial; in the primary health care facilities nurses tended to be permanent and experienced, in contrast to the short term junior doctors. Many others, however, doubted their ability to engage with such a system, although their reservations related to nurses’ perceived lack of familiarity with smart phone technology rather than ability to use telemedicine per se. A previous study analysing telemedicine referrals from nurses, physiotherapists and other health care workers, found little difference between their responses and those of physicians [29, 44], however, the position of allied staff, their professional standing and potential for role extension varies widely between different countries.

Another related issue that was raised pertained to the idea of hierarchy extending not only to individuals but to institutions and specialties. Some participants queried the relative merits of systems which solely included tele-experts attached to the tertiary burn unit, as opposed to those employing experts from a variety of secondary and tertiary level facilities. Others raised the question of specialty, and on what grounds somebody would be deemed and remain an ‘expert’ within such systems, in terms of credentials or experience.

*“I think that it might be, it’s something that, that would need to be quite carefully, trod around, in terms of who’s going to be the experts... I just think it needs to be strategically done. And I was thinking maybe, I mean, maybe there could be like an, exam, or something, some sort of qualification before you actually are allowed to be an expert.”-ID.3*

In a system which combines the talents of two specialties, as in many trauma and emergency situations, this is a question which may require consideration at implementation. Simultaneously however, potential was seen to reduce a professional gap at this expert level; greater collaboration between specialties and institutions could enhance partnerships, and foster deeper mutual respect. In general it was perceived that telemedicine could be used to counteract negative effects of a hierarchical system, but would neither dispel nor entrench the hierarchical structure itself.

#### Experts as gatekeepers

A connected discussion involved issues of communication and trust, in a system where the expert can be viewed as the ‘gatekeeper’ to scarce resources. Related to this was the proposed benefit of images adding ‘proof’ or ‘evidence’ to a query. Many of the experts suspected an exaggeration or underplaying of the severity of cases during telephone consultation by POC staff, in order that the patient would fit the criteria for referral.

*“when a burn patient comes in, is this patient going to get that golden bed....like what is it about this patient that I can package them in a way, and sell them in a way to get them into that bed”-ID.11*

Many experts expressed empathy towards the motivation behind such misrepresentations of a patient’s condition. Simultaneously, however, many viewed this ‘tactic’ as furthering the crisis in referral and resources, leading to patients directed to inappropriate levels of care. It was thought that the validity and that an app could bring was essential for appropriate disposition, but also for long term relationship building between colleagues and institutions as this ‘evidence’ component eroded previous suspicion and distrust. The possibility was raised however, that such systems could be underused at POC for this exact same reason.

Here, the key elements for interaction with such a technology to effectively elevate practice related to improved relationships, greater transparency of communication a more formalised access to colleagues and definition of their roles.

### Physical divides within the trauma journey

One of the greatest divides related to tangible gaps; the physical distance between where trauma cases first present to medical services and where they can best be treated, between the resources and facilities of the primary health care facilities and the specialist tertiary units, and potentially between the experience and skills of their respective staff.

Here, huge potential for diagnostic telemedicine systems to bridge these gaps was anticipated. Suggested benefits pertained to a greater understanding and appreciation of the difficulties and roles between the primary health care facilities and tertiary units, better information exchange leading to enhanced ability to advise, and greater confidence in doing so. Consequential to these crucial enhancements was better initial treatment so vital in trauma and emergency, greater adherence to referral criteria ensuring the right patients selected for transfer, and an overall uplifting of the service. Challenges still remained however related to how new pathways for referral would fit into existing systems, and how inexperienced doctors would respond to greater information. However, the key anticipated areas of potential improvement relating to formalisation of information exchange, and a corresponding increase in the experts’ confidence in their ability to advise were strongly echoed by many of the participants.

Much of the specific information gleaned within this category is beyond the scope of this paper, and has been discussed in greater detail in another work relating to specialists’ expectations of image based teleconsultation [45].

## Discussion

Directly addressing the tele-experts and taking a broad approach to the subject matter provided both corroboration of previous findings and extension of existing knowledge. While limited studies from HICs have found positive potential for Trauma and Emergency telemedicine [7–9], others have suggested prerequisite conditions of strong infrastructure for success, or where this does not exist, introduction through ‘simple’ functions such as video conferencing [46]. The view of the South African experts would seem to strongly disagree with this. While acknowledging the additional demands resource constrained settings put on such systems, the expert view appeared overall optimistic.

Many of the findings touched on interdependencies of different users and how evolution will occur organically in response to new technology. Improved information exchange allows experts to advise better, this allows POC to treat better, patients arrive to tertiary care more optimised allowing experts better treatment options, affording them, too, the capacity to improve the standard of care they provide.

Telemedicine’s inherent ability to span borders and contexts means much knowledge is transferable even between vastly differing contexts; the main areas of impact presented here indicate that many of the tele-experts’ concerns and expectations correlate with those of POC users, and those found in HIC systems. Areas where consensus was found on opportunities, such as education and relationship building, have been previously cited as strong benefits of telemedicine [10]. It is important to note, however, that the experts - would be educators and participants in these relationships - agree that these findings apply to the acute resource restrained context, and are keen to engage with these functions. Areas of lower consensus in the study, notably the role of feedback and the inclusion of allied health professionals, perhaps pinpoint areas needing greater attention on a case by case basis for implementation.

### Formalisation and the effect on ‘gaps’ in the system

It is hoped that the interviews, exploring both daily working practice, and relationships with technology such as apps, revealed how this technology could be best utilised to reduce some of the challenges of working in such settings.

Running across all themes was an idea of ‘gaps’ in the system creating barriers. Within the ‘gold standard practice vs achievable practice’ theme, we elucidate gaps such as those in staff experience from POC to referral center, and resources available, gaps between the desire to care and the ability to do so, and from the Power and Personality category the effects of distance on relationship, as well as status and seniority.

A formalisation of processes and practices emerged as an important aspect in the latent potential for telemedicine to bridge gaps of inequality in the trauma and emergency system, with aspects relating to the themes which were uncovered. Some are more apparent, the formalisation of queries through a technology interface means data is provided efficiently and systematically, facilitating the exchange of information and advice. The formalisation of data storage is effective for managing cases in continuous care, research, and legal documentation. Further, formalised systems may standardise care, so inherent inequalities are reduced.

Less obvious perhaps are the benefits of formalisation of relationships and interpersonal exchanges, relating to some of the ideas explored under the ‘power and personality’ theme. Such formalisation can reduce intimidation founded in hierarchy and open communication channels, increasing options for new professionals who lack the contacts to approach colleagues informally. Awareness of the option to seek help is enhanced, and the resilience and self-reliance required to manage the professional and personal challenges described in such a setting could be diminished. More, a formalisation of the role of the experts and access to them enhances their capacity to provide essential education and support.

One possible key benefit then of such apps, may be to harness the potential of technology to formalise processes, reducing gaps and inequalities within systems. Our data suggested the potential for this process through technology in this setting related to 4 key areas of impact– increased access, the ability to advise, education and relationships. Such information could allow focus on these aspects, which if considered forefront in development and introduction of new technologies, could increase their positive impact on practice, and thus their acceptability and uptake.

The importance attached to formalisation is perhaps unsurprising within medical culture and the trauma and emergency environment where accuracy and attention to detail can be critical. Respecting pre-defined ‘rules’ within medicine could prove key in technology implementation, and as previously noted, telemedicine needs to work within the system for maximum effect, rather than aiming to change it.

It is also acknowledged however, that informal processes developed organically as solutions to problems, and in some cases formalisation could have the opposite of the desired effect on the issues it seeks to address. As with any change, formalisation can be opposed initially, hit teething problems and inevitably involve ‘trade off’ in some areas rather than straight out gains, but these were concessions which most experts seemed willing to entertain.

**Fig. 2:**
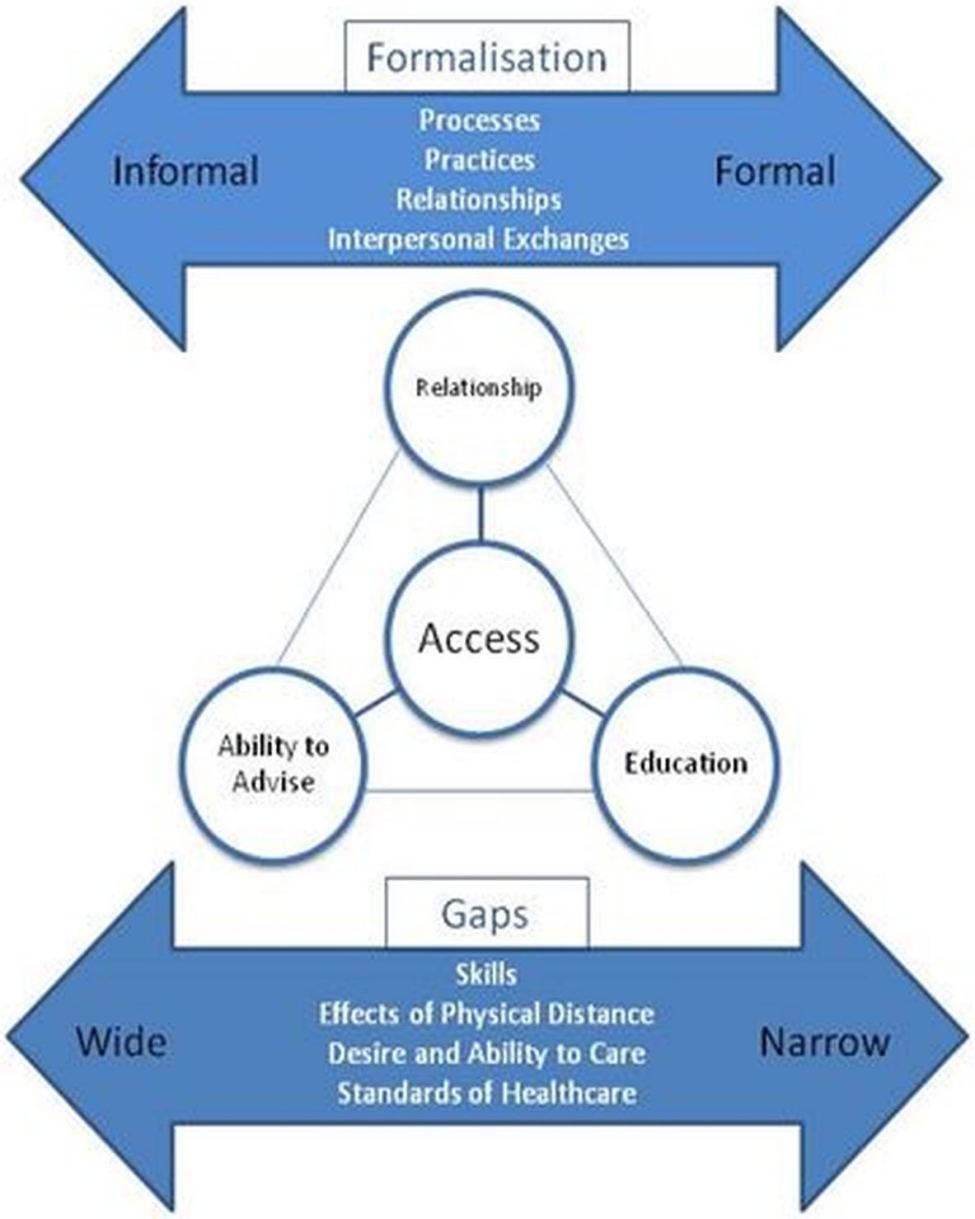
Graphical representation of findings

Figure 2 illustrates the proposed main opportunities of our results, interplay between effects and outcomes, and how key themes and categories relate.

### Implications for implementation of telemedicine systems in trauma and emergency

The tensions highlighted in the study are presented in the hope that, in implementation of design of such systems, benefit can be gained from prior consideration of these factors. In many examples, there is no given solution as the context of the locality will widely impact positive resolutions to the challenges highlighted. For example, issues around ethics and sensitivities will obviously vary widely according to cultural context. In some settings, the very idea of photography will be a barrier to overcome, in others, gender issues may require consideration. In cultures with a high use of internet technology, suspicion around the use and secure storage of data may prove a growing concern.

In some areas however, recommendations can be made addressing certain recurring concerns. Looking at the ideas within the theme of ‘power and personality’ a number of opportunities exist to address challenges and enhance interpersonal relations; training should underline the experts’ acknowledgement of the challenges at POC and demonstrate the experts’ willingness to participate and reduce stress around communications for their junior colleagues. It is also interesting that experts questioned how ‘the experts’ of such systems would be defined, and remain current. Practical examples could include a test for inclusion as a system expert, with great transparency, giving reassurance to both experts and POC users of the advice provided. This will also be particularly pertinent in assessing the role of allied health profession and nursing staff.

Other practical solutions can be proposed relating the challenges outlined in the ‘gold start practice vs achievable practice’ theme. In a system where remote consultation already occurs via informal solutions, these technologies, must not only impress, but compete with informal alternatives. To address the inevitable competition from apps such as WhatsApp, it is suggested that unique aspects are developed and highlighted at implementation, such as the ability to store and organise case data, the inclusion of additional diagnostic tools, and the legal benefits of patient confidentiality. In terms of addressing the issues around sensitivity and care, and the dehumanisation associated with the greater use of technology, the technology itself can be used to counteract such effects. For example, the suggested use of ‘pop-up prompts’ within tele-medicine systems, or the idea of using a thumb print onto the screen to demonstrate patient consent.

The differences in focus between burns and emergency specialists are interesting to consider. The differently weighted emphases perhaps points to potential gains at the conceptual design and implementation stages of such tools through addressing both specialties, and further to consider which specialist input is best sought when addressing particular features. For example; heeding emergency physicians’ warnings on system issues relating to speed, reliability and user friendly design may include incorporating ringtone rather than text alerts, delivery reports, and installation on personal devices or static units within the department which could not be lost or misplaced. Simultaneously, seeking subspecialty expert advice on technical details of how best to capture the most pertinent specific injury details, such as in the case of burns, a circumferential component or airway involvement.

As stated at the outset, a complex area of this study was the many subspecialties that are called on within the field of trauma and emergency, burns being just one. This raises a major challenge; each subspecialty is very diverse and specifics such as those above can be unique to each, but from the physician point of view, maintaining countless apps each pertaining to a different subspecialty would be challenging. Rather than being a limiting factor, perhaps key is to identify such specifics and envisage systems in trauma and emergency which, whilst functioning as one streamlined application, contain areas of input tailored to each specialty and are thus adaptable to the emergency at hand. Further work in applicable subspecialties would be required to identify common areas and those requiring particular input, and then to collate this information. The positive results found in acute burns, however, suggest that other subspecialties may stand to benefit similarly, and thus work in this area could be extremely productive.

### Strengths and limitations

The capture of only one subspecialty within trauma and emergency, and only the expert user view are limiting factors to the study; comparisons with the view of a ‘referrer user’ and with experts of another trauma and emergency subspecialty would add interest and validity. It is hoped however that the method allows for transferability of results to similar contexts which can relate to the challenges of limited expert knowledge on site, and great distances between point of care and referral facilities. Methodologically, the possibility of project bias must be highlighted, as the interviewees, as experts within the project, may have a vested interest in its success and positive portrayal. As noted above, some had previous knowledge of the app, had contributed in some way to various elements of the conceptual design or participated in user training. At this early stage in the process prior to the emergence of concrete issues, an element of ‘over positive’ or ‘wishful’ thinking may emerge and positively skew results in favour of the possible impacts of the app. Conversely, experts may also have the incentive to over emphasis possible issues, with the objective of resolving any such problems prior to implementation. However, the wide exploration of the challenges and positives points to a willingness for free thinking on the subject, and critical analysis of the possible problems to be encountered.

Given the small sample size, participants may have feared identification through their opinions, despite every effort being made to protect anonymity. Further, the possibility of social desirability bias, and of elite participants speaking for an institution or ideal rather than indulging a personal view cannot be excluded [40].

## Conclusions

Experts perceive many potential implications of the introduction of mHealth support for diagnosis and advice, including enhanced opportunity to fulfil their role as experts, and ensuing positive impact for other users and patients. It was believed that aspects of such apps could improve provision of trauma and emergency care in a resource constrained setting, in terms of reduced delays, quality, and outcomes. Such settings can bring exacerbated and extended challenges to acute trauma care telemedicine however, and in the view of expert users, there are specifics of the environment which would demand further and careful consideration for successful implementation – time pressure, intensity, and the wide range of subspecialties to be considered.

The overriding view of the tele-experts was that there is a place for clinical diagnosis telemedicine systems within trauma and emergency. Although special considerations are required, diagnostic telemedicine has the potential to bridge gaps in healthcare provision, which translate to inequalities in care in the current system.

## Additional file

Additional file 1: Interview Guide. (DOCX 33 kb)

## Abbreviations

App: Mobile application software
DALYs: Disability-Adjusted Life Years
EM: Emergency Medicine
HICs: High-income countries
LICs: Low-income countries
LMICs: Low- and middle-income countries
POC: Point-of-care
SA: South Africa
