# Supplementary material for: What do emergency medicine and burns specialists from resource constrained settings expect from mHealth-based diagnostic support? A qualitative study examining the case of acute burn care
Source: BMC Med Inform Decis Mak. 2018 Aug 1;18:71. doi: 10.1186/s12911-018-0647-1 (PMC6090944; doi:10.1186/s12911-018-0647-1)
Supplement: Supplementary file 1 — Interview Guide. (DOCX 33 kb) [file 12911_2018_647_MOESM1_ESM.docx]

**Additional file 1: Interview Guide**

**Work experience in the health sector and with using telemedicine**

We are discussing today a new App which seeks to benefit healthcare by exploiting the use of telehealth, or telemedicine. This kind of technology can obviously take many forms, but we are primarily interested in hearing about your experience of image based telemedicine systems. We would like to begin however, by hearing about your experiences in health care in general.

1. Could you tell me a bit about your current role and how long have you been working there / in this health care facility?
2. Where did you work before here? / Would you describe for me your career path leading up to this point?
3. In your previous roles, have you ever come across or used what you think of as image based telemedicine before?
4. When did you first encounter this particular Telehealth project (mHealth for burns diagnostics and care South Africa)?

**Experiences of diagnosing burns patients at bedside and remotely**

Before we discuss the App we would like to understand more about the context and your current working practice as an expert. So for these first questions we’d like you to tell us how things work at the moment, without the App.

*Assess fresh burns*

1. In your current practice, do you regularly assess patients with fresh burns at point of care?

*Challenges assessing*

1. When you assess a new patient yourself, at bedside, could you tell me, what are the most challenging aspects of assessing a new patient?

*Remote advice*

1. a) We understand that giving advice remotely on burn patients occurs in your work. Is that correct?

b) Is there a standard procedure or protocol for this, or how does it happen otherwise?

c) We believe a part of your work can also involve discussing referrals. So we would like to know on what grounds the decision is made to refer a patient for further treatment?

d) In your experience is there discordance between the clinical information you receive remotely, say over the phone, and the clinical information you observe when the patient arrives to your care? If yes, how often?

e) When you discuss a case in this way but it is decided that the patient does not need referral, or cannot be referred at this time, what happens? Do you give, or are asked to provide management advice for the treatment of the patient in the remote location?

**Experiences and views on the new App**

Thank you. We would now like to ask about your thoughts or pre-apprehensions about the introduction of the App, so in the following questions we’d like to ask you to imagine the situation you envisage once the App has been implemented as a tool in your work place.

*Changes from the App*

1. Could you tell me about what sort of effects do you think the App will have on the processes you have described, and your advisory role?

*Daily workload*

1. a) When first introduced, do you think the App is going to influence your workload or daily tasks? Say during the first 6 months?

b) And what about it the long term? Could it have any effect on work burden or tasks in a long term capacity?

*Colleagues*

1. Do you anticipate any changes in your relationship to remote clinicians, considering how you communicate with them now and how you will communicate with the new App?

*Patients*

1. a) Can you foresee any positive factors or outcomes through introduction of the App, from their points of view?

b) Do you see any risks or disadvantages for patients with the introduction of the App?

*Healthcare System*

1. a) Can you foresee any potential challenges in the health care system in relation to the implementation of the App – or of similar systems?

b) Do these changes (as mentioned) have any implications for your role as expert in the broader healthcare system?

*Technology and usability*

To participant who have been introduced to the App before the interview:

1. a) From your experience with using the App so far or from your introduction to it, do you anticipate any issues relating to the technology?

b) And again from your experience so far, do you anticipate any issues relating to the usability of the App, for yourself or others?

*Feedback*

1. Some telehealth systems are now providing the capacity for users to receive feedback on cases they have advised for. What are the advantages and disadvantages as you see them?

*Capture outcome*

1. Finally, being able to assess the impact of the introduction of an App is important. Do you have suggestions on how to capture this?

**Final Questions**

1. Considering everything we have spoken about today:
2. What do you consider the most important factors for its success? Could you give us 2 or 3 of the most positive aspects?
3. What do you see as the weaknesses of such a system that could lead to its failure? Could you give us 2 or 3 of what you consider the most negative aspects of the project?
4. Are there any final points you would like to add on any aspect of the subject, or anything you think we have not covered?

**Close**

Many thanks for your time, your participation is invaluable for the study.

We will contact you once we have transcribed the interview to arrange a time for a brief respondent check to make sure we have understood your views correctly.
